# Self‐compassion and psychological distress in chronic illness: A meta‐analysis

**DOI:** 10.1111/bjhp.12761

**Published:** 2024-11-07

**Authors:** Rebecca Baxter, Fuschia M. Sirois

**Affiliations:** ^1^ Chesterfield Royal Hospital NHS Foundation Trust Chesterfield UK; ^2^ University of Sheffield Sheffield UK; ^3^ Durham University Durham UK

**Keywords:** chronic illness, meta‐analysis, psychological distress, self‐compassion, stress

## Abstract

**Objectives:**

Self‐compassion is a positive psychological factor linked to improved physical and psychological outcomes across different chronic illness populations. However, the extent to which self‐compassion contributes to reduced distress across different conditions or as a function of participant factors is not clear. The current meta‐analysis aimed to quantify the association between self‐compassion and psychological distress in different chronic illness populations and evaluate the factors that moderate this association.

**Methods:**

A systematic search of three electronic databases identified research reporting associations between self‐compassion and psychological distress in chronic illness. A random effects meta‐analysis was conducted to evaluate the association between self‐compassion and psychological distress. Moderator analyses were conducted for sample characteristics and distress types. A bespoke tool evaluated study quality.

**Results:**

Searches yielded 51 eligible studies with 57 effect sizes. Meta‐analysis revealed that self‐compassion was negatively associated with psychological distress (*r* = −.516; 95% CIs [−.55, −.48]; *p* = .000). Moderator analyses were significant for distress type and chronic illness group, with effects being largest for stress and neurological conditions. Effects did not vary by sex, age or illness duration.

**Conclusions:**

Findings from this first comprehensive investigation of the link between self‐compassion and distress in chronic illness highlight the protective role of self‐compassion for chronic illness populations. These results lay the foundation for further research into understanding the processes that link self‐compassion to lower psychological distress, and that examine the effectiveness of self‐compassion interventions in chronic illness populations, to further advance knowledge and inform practice in this area.

## INTRODUCTION

Chronic illness is a broad term with variances in its definition across different professional bodies and countries. However, it is generally agreed that chronic illness persists across time and significantly impacts day‐to‐day life (Bernell & Howard, 2016; Moss‐Morris, 2013). This term encompasses a broad range of persistent medical conditions, such as cardiovascular diseases, cancers, chronic respiratory diseases and diabetes are the main types of chronic illness (World Health Organization [WHO], 2022), as well as long‐term neurological disorders such as epilepsy and dementia (Feigin et al., 2019), chronic pain disorders including arthritis (Fayaz et al., 2016), inflammatory diseases (Ford et al., 2011) and Human Immunodeficiency Virus (HIV) (Deeks et al., 2013). Estimates suggest that approximately 15 million people in England are living with a chronic illness (Department of Health [DoH], 2012).

Chronic illnesses are generally incurable and often involve symptom management and increasing disability (Panjwani & Revenson, 2020), making them akin to living with a chronic stressor in terms of their psychological impact. Not surprisingly that chronic illness can have a profound impact on psychological well‐being (De Ridder et al., 2008) and contribute to anxiety, depression and stress, which in turn can increase disease burden (Gold et al., 2020; Naylor et al., 2012). Understanding the factors associated with distress in this population is therefore crucial for improving disease management.

Self‐compassion is one positive psychological quality linked to improved physical and psychological outcomes across different chronic illness populations. Neff (2003a) conceptualizes self‐compassion as responding to personal suffering and failures in an accepting and mindful way whilst recognizing the common experience of human suffering (Neff, 2003a). Research has highlighted that self‐compassion is linked to psychological well‐being in various chronic illness populations and is proposed to free up emotion regulation resources, alter cognitive appraisal and improve coping (Sirois, 2015; Sirois, Molnar, & Hirsch, 2015). However, the magnitude of the association between self‐compassion and psychological distress in this population, and the factors that may amplify or attenuate this link remain unclear. Such knowledge can have important implications for interventions used to treat psychological distress in the chronic illness population and for future research examining self‐compassion. The current meta‐analysis aimed to provide important insights into the association between self‐compassion and psychological distress in the context of chronic illness.

### Psychological distress and chronic illness

Psychological distress is an umbrella term for an emotional state that often includes symptoms of stress, anxiety and depression, experienced by an individual in response to a specific situation or trigger (Ridner, 2004; Viertiö et al., 2021). It is transient in nature, varies in intensity, and may dissipate when the individual's context changes or they adapt to the stressor. However, struggles to cope with the stressor can worsen distress, and become a diagnosable mental health condition such as anxiety, depression, or Post‐Traumatic Stress Disorder (Phillips, 2009; Sirois & Owens, 2021).

The significant adjustments to daily life, functional limitations (Sirois, Kitner, & Hirsch, 2015), reduced independence (Mistretta & Davis, 2022) and impact on sense of identity (Panjwani & Revenson, 2020) that accompany living with a chronic illness can increase vulnerability to psychological distress. Indeed, physical health‐related stressors can directly contribute towards depression in those with chronic health conditions (Warner et al., 2019), and the unpredictable nature of chronic illness can lead to feelings of anxiety about the future (Panjwani & Revenson, 2020).

The impact of psychological distress on health outcomes in those with chronic illness is well‐documented and highlights the need to better understand protective factors. Increased functional impairment, poor health behaviours and symptom management, reduced treatment adherence and lack of support through social withdrawal (Homan & Sirois, 2017; Katon & Ciechanowski, 2002; Martin et al., 2005), are known routes through which distress can impact health outcomes. For chronic conditions that involve inflammation, stress and especially chronic stress, can further exacerbate dysregulation of inflammatory processes, and disease symptoms (Cohen et al., 2012, 2007; Maunder, 2005). Stress is therefore a particular issue for chronic illnesses that involve underlying inflammatory processes, such as arthritis (e.g. Evers et al., 2013), cardiovascular disease (e.g. Rod et al., 2009) and inflammatory bowel disease (e.g. Jaghulta et al., 2013).

### Self‐compassion and distress in chronic illness

The three bipolar components of self‐compassion (Neff, 2003a) reflect key qualities that can be protective against the distress experienced from living with a chronic illness. Self‐kindness versus self‐judgement involves being understanding rather than self‐critical when faced with personal difficulties or shortcomings. Common humanity refers to viewing suffering and failure as part of the human condition, rather than something isolating and unique to the individual. Mindfulness involves taking a balanced approach to negative thoughts and feelings, rather than over‐identifying with them or trying to avoid or repress them (Neff, 2003a, 2003b). In the context of health, the three components of self‐compassion operate synergistically to positively impact health through improving efficacy beliefs, supporting emotion regulation and enhancing adaptive coping strategies which can in turn reduce stress (Sirois, 2023).

Consistent with this view, research has highlighted various ways in which self‐compassion is beneficial for coping with the distress associated with chronic illness. The three facets of self‐compassion can positively influence how difficulties are appraised, which in turn predicts more adaptive coping and less stress (Sirois, Molnar, & Hirsch, 2015). Self‐compassion contributes to positive appraisals of chronic illness‐related difficulties through reducing the tendency to catastrophize and ruminate over them (Purdie & Morley, 2015; Wren et al., 2012), instead promoting a more accepting view of difficulties as being part of life (Costa & Pinto‐Gouveia, 2011; Morgenroth et al., 2022; Pinto‐Gouveia et al., 2015). Positively reframing and accepting chronic health‐related difficulties supports the use of adaptive coping strategies that reduce stress, rather than maladaptive ones that contribute to psychological distress (Allen & Leary, 2010; Neff et al., 2007; Sirois, Molnar, & Hirsch, 2015).

### Current study

Previous narrative reviews have highlighted the link between self‐compassion and psychological distress in groups of individuals with specific types of chronic illness diagnoses (e.g. Hughes et al., 2021; Longworth, 2020; Misurya et al., 2020). Yet the magnitude of this association has yet to be quantified, and the factors that might attenuate or amplify this association have not been systematically investigated across chronic illness populations. Such information is crucial for understanding the optimum delivery of self‐compassion‐based psychological therapies in this population.

Accordingly, our meta‐analysis investigated the strength and direction of the association between self‐compassion and psychological distress in chronic illness populations, with the expectation that self‐compassion would be associated with less psychological distress. To better understand the factors that can influence this association, we conducted moderator analysis focusing on potential methodological and sample‐related factors.

Research examining the association between self‐compassion and multiple types of psychological distress have reported variations in effect sizes as a function of the type of psychological distress (e.g. Costa & Pinto‐Gouveia, 2011), suggesting that distress type may moderate this association. Testing this moderator therefore has implications for the relevance of self‐compassion interventions for dealing with different types of distress. Because there is some evidence that the association between self‐compassion and psychological distress varies between different chronic illness populations (e.g. Pinto‐Gouveia et al., 2014), we examined illness type as a potential moderator. Illness duration was also included as an additional exploratory moderator that was not included in pre‐registration of the meta‐analysis. Research has consistently found that males have higher levels of self‐compassion than females (Yarnell et al., 2015, 2019), and self‐compassion increases with age (Homan, 2016; Toth‐Kiraly & Neff, 2021). Accordingly, we expected that the association between self‐compassion and psychological distress would vary as a function of sample age and proportion of females in the sample.

## METHODS

### Protocol registration

This meta‐analysis was pre‐registered on PROSPERO which can be accessed via the following link: https://www.crd.york.ac.uk/prospero/display_record.php?ID=CRD42023387333.

### Literature search

Relevant literature was identified through searching three electronic databases (Scopus, PsycInfo and Medline via Ovid), along with the first 10 pages of Google Scholar, on 16th January 2023, and then again on 12th March 2024. The decision to search only the first 10 pages of Google Scholar was based on the relevance of results returned by Google Search's PageRank algorithm which ranks most relevant content first, making results beyond the first 100 hits less profitable in terms of identifying potential studies, as well as less feasible (Briscoe et al., 2023), especially when resources are limited. Indeed, research has found that unique results from searching Google Scholar are found within the first 100 studies (Briscoe et al., 2023).

Search terms appropriate to each database were developed using an iterative scoping process (Table 1). Given the breadth of the concept and the variations in definition across the literature (Phillips, 2009), for the purpose of this meta‐analysis, psychological distress included both disorder‐specific terms and symptoms such as ‘anxiety’, ‘depression’ and ‘stress’ as well as broader concepts such as ‘psychological difficulties’ to capture these aspects of the definition. Similarly, broader terms for ‘chronic illness’ were included, as well as disease‐specific terms. Terms were searched for within titles, abstracts and keywords. MESH terms were included where appropriate. Reference lists of identified papers were searched for additional relevant papers, and forward reference searches were completed to identify relevant papers which had referenced papers already identified for inclusion since their publication. Grey literature was included to increase methodological rigour (Conn et al., 2003; Hopewell et al., 2007).

**TABLE 1:** Table showing search terms used in literature search (OR used within columns and AND across columns).

Self‐compassion	Psychological distress	Chronic illness
“self‐compassion”, “compassion”	“depress*”, “anxiety”, “stress”, “post‐traumatic stress”, “PTSD”, “psychological distress”, “distress”, “mental health difficulties”, “psychological difficulties”	“chronic* ill*”, “chronic disease*”, “physical health condition*”, “long term health condition*”, “medical condition*”, “cystic fibrosis”, “fibromyalgia”, “rheumatoid arthritis”, “cancer”, “dementia”, “parkinson's”, “human immunodeficiency virus”, “acquired immune deficiency syndrome”, “heart disease”, “multiple sclerosis”, “chronic fatigue”, “epilepsy”, “asthma”, “chronic pain”, “chronic obstructive pulmonary disease”, “inflammatory bowel disease”, “irritable bowel syndrome”, “diabetes”, “endometriosis”

### Inclusion criteria and data extraction

Studies meeting the following criteria were included in the meta‐analysis; (1) individuals were age 18 and over with a chronic illness diagnosis, (2) included a validated measure of self‐compassion and psychological distress, (3) utilized quantitative or mixed methods, (4) available in English language, (5) data available for the association between self‐compassion and psychological distress or available upon request, and (6) full‐text was available. Studies were excluded if they; (1) did not meet inclusion criteria, (2) were an editorial, letter, discussion paper, guidance document, conference paper or book review or were a systematic, scoping or literature review.

Effects were extracted as Pearson's *r* as they were the most frequently reported effect. For longitudinal or intervention studies, the *r* value for the baseline association between self‐compassion and psychological distress at baseline was recorded. Where this data was not available the effect size at the next closest time point was extracted.

Essential information about the sample (*N*, % female, age, study country, % Caucasian) and study (publication status, study design) were also extracted. Additional moderator information was recorded for each study, including type of psychological distress, type of chronic illness, and illness duration. A second reviewer (a Trainee Clinical Psychologist) extracted data from a third of the papers selected at random.

### Quality analysis

We assessed the quality of the studies included using a tool adapted from the Appraisal Tool for Cross‐sectional Studies (AXIS; Downes et al., 2016) by Sirois and Owens (2021) following the suggestions by Quintana (2015). The tool consisted of 11 criteria of relevance to cross‐sectional research, with papers given a score of ‘1’ for each criterion they meet or ‘0’ if they fail to meet the criterion (Appendix A: Data S1). Scores were summed, with a score of five or less indicated low quality, six to eight indicated moderate quality, and above eight indicated high quality (Sirois & Owens, 2021). A third of the papers were randomly selected and checked by a second rater (a Trainee Clinical Psychologist), with disagreements resolved through discussion.

### Analyses

The magnitude of the association between self‐compassion and distress was estimated in a random effects meta‐analysis conducted with the Comprehensive Meta‐Analysis software (CMA; Version 3, Borenstein et al., 2013). CMA converts the correlation co‐efficients to Fisher's *Z*‐scores before meta‐analysing them. We interpreted effect sizes using Cohen's (1992) criteria, with *r* = .10 representing a small effect size, *r* = .30 a medium and *r* = .50 a large effect size. Where studies reported multiple effects, an average effect size for overall distress was calculated to avoid over‐inflating the magnitude of the effects (Card, 2012). Similarly, where studies only reported effects for individual subscales of the SCS or a two‐factor variation of the SCS, an average effect size was calculated.

#### Heterogeneity

*Q* and *I* ^*2*^ statistics were calculated to test for and quantify study heterogeneity and determine whether moderator analyses were warranted (Card, 2012). A significant *Q* statistic indicates the presence of heterogeneity among the pooled effect sizes, beyond that which can be explained by sampling error (Borenstein et al., 2010). The *I* ^*2*^ statistic indicates the percentage of variability that is not due to sampling error, with 25% indicating low, 50% indicating moderate and 75% indicating high heterogeneity (Higgins et al., 2003).

Moderator analyses were conducted to investigate sources of variance when tests indicated significant heterogeneity. Subgroup analyses were planned for categorical data (outcome type and illness type) when there were three or more effect sizes per subgroup as recommended by Card (2012). Meta‐regressions were planned for continuous moderators (age, gender, percentage female and illness duration) when there were at least 10 studies.

#### Sensitivity analyses

Sensitivity analyses were conducted to examine whether inclusion of studies with methodological differences impacted the overall results of the meta‐analysis. The pooled effect size produced following sensitivity analyses was then compared to the pooled effect size produced prior to studies being removed.

#### Publication bias

We took a multi‐pronged approach to assess the risk of this publication bias.

A fail‐safe *N* (Rosenthal, 1979) was calculated to estimate how many studies with non‐significant associations could be added to the observed pooled effect size, before the *p* value would become insignificant. An *N* of at least *5 k* + 10 (where *k* is the number of samples in the analysis) is considered sufficiently high to suggest low likelihood of publication bias (Rosenthal, 1979). Duval and Tweedie's (2000) trim‐and‐fill method assessed whether studies being removed (‘trimmed’) or added (‘filled’) to create symmetry around the mean would alter the overall findings. This was judged visually by comparing the funnel plot displaying the values from the studies included in the meta‐analysis, with the funnel plot displaying the ‘trim‐and‐fill’ values (Duval & Tweedie, 2000). Then, Egger's regression test was conducted to statistically measure the degree of asymmetry present in the funnel plot (Sterne & Egger, 2005), with a significant value being suggestive of publication bias (Egger et al., 1997).

## RESULTS

### Study characteristics

**FIGURE 1:**
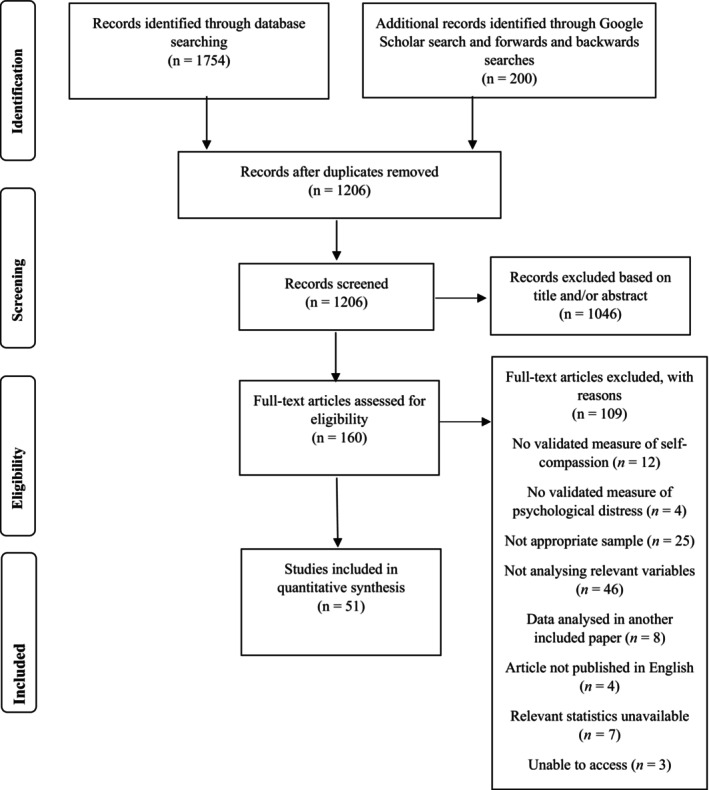
PRISMA diagram of study selection process.

Fifty‐one studies met inclusion criteria (Total *N* = 15,424), resulting in 57 effects being included in the meta‐analysis (see Figure 1 for PRISMA flow chart). The pool of included studies investigated a broad range of chronic illnesses (see Table 2). All but one study (Harrison et al., 2017) used variations of the Self‐Compassion Scale (SCS; Neff, 2003a; See Table 3). Thirty‐eight studies measured symptoms of depression using nine different measures, 25 studies measured symptoms of anxiety using 11 different measures, 15 studies measured stress using five different measures, four studies measured diabetes distress using four different measures, four studies measured negative affect using the same measure, and five studies measured overall psychological distress using four different measures. Eighteen studies used multiple measures of distress; therefore 38 of the 57 effects reflected the combining of effects (See Table 3).

**TABLE 2:** Characteristics of the 51 studies included in the meta‐analysis.

Author (year of publication)	Analysed sample size (*N*)	Country	Study design	Diagnosis	Mean age	% female	Illness duration (years)
Abdollahi et al. (2020)	210	Iran	Cross‐sectional	Breast cancer	43.2	100	9.2
Baker et al. (2019)	270	UK	Cross‐sectional	Epilepsy	–	76.2	–
Campbell et al. (2022)	108	Australia	Experimental	Multiple sclerosis	–	–	–
Carvalho et al. (2018)	231	Portugal	Cross‐sectional	Chronic pain	48.51	100	–
Carvalho et al. (2020)	49	Portugal	Cross‐sectional	Chronic pain	50.49	100	–
Carvalho et al. (2022)	49	Sweden	Experimental	Various	43.2	85.7	–
Costa and Pinto‐Gouveia (2011)	103	Portugal	Cross‐sectional	Rheumatoid arthritis and chronic pain	60.22	79.6	–
Davey et al. (2020)	420	UK	Cross‐sectional	Chronic pain	48.2	74	12
Day (2019)	278	UK	Prospective	Multiple sclerosis	46.33	80	11.41
Eccles et al. (2023)	130	UK	Cross‐sectional	Parkinson's disease	64.68	56.9	5.21
Emmerich et al. (2020)	316	Germany	Longitudinal	Chronic pain	45.83	90.5	–
Friis et al. (2015)	100	New Zealand	Cross‐sectional	Diabetes	57.6	65.6	16.7
Gedik and Idiman (2020)	89	Turkey	Cross‐sectional	Multiple sclerosis	39.78	75.3	7.26
Harrison et al. (2017)	70	Canada	Cross‐sectional	Chronic obstructive pulmonary disease	70.8	66	8.8
Hirsch et al. (2021)	1218	Austria	Cross‐sectional	Fibromyalgia, Rheumatoid arthritis, Osteoarthritis and Ankylosing spondylitis	58	52	–
Ho et al. (2022)	127	China	Cross‐sectional	Colorectal cancer	63.8	58.3	–
Houston (2022)	147	UK	Cross‐sectional	Chronic fatigue syndrome/Myalgic encephalomyelitis	40.98	87.8	11.63
Kauser et al. (2021)	114	UK	Cross‐sectional	Cystic fibrosis	32.32	49.1	–
Kelliher‐Rabon et al. (2022)	S1: 419 S2: 235	USA	Cross‐sectional	S1: Fibromyalgia S2: Cancer	S1: 47.66 S2: 61.28	S1: 95.7 S2: 63.4	–
Kemppainen et al. (2013)	1969	USA	Cross‐sectional	Human immuno‐deficiency virus	45.11	27.2	12.93
Kılıç et al. (2022)	116	UK	Cross‐sectional/ Longitudinal	Type 2 diabetes	–	–	–
Matos‐Pina et al. (2022)	223	Portugal	Cross‐sectional	Various	38.53	87.9	–
Morgenroth et al. (2022)	45	Germany	Cross‐sectional	Heart failure	60.5	20	8.71
Morrison et al. (2021)	176	UK	Cross‐sectional	Type 2 diabetes	–	31.8	–
Ogueji (2021)	832	Nigeria	Cross‐sectional	Human immuno‐deficiency virus	38.86	100	–
O'Loughlin et al. (2020)	111	USA	Cross‐sectional	Human immuno‐deficiency virus	42.8	46.1	13.7
Pinto‐Gouveia et al. (2014)	S1: 68 S2: 63	Portugal	Cross‐sectional /Longitudinal	S1: Various S2: Cancer	S1: 51.55 S2: 54.04	S1: 75 S2: 82.5	–
Potter et al. (2020)	144	Canada	Cross‐sectional	Irritable bowel syndrome	21.65	80.6	–
Purdie and Morley (2015)	60	UK	Experimental	Chronic pain	46.9	76	13.9
Rawlings et al. (2023)	65	UK	Cross‐sectional	Pulmonary hypertension	56.91	81.5	8.23
Santerre‐Baillargeon et al. (2018)	48	Canada	Cross‐sectional	Vulvodynia	26.83	100	6.15
Schellekens et al. (2017)	88	Netherlands	Cross‐sectional	Lung cancer	62.8	33	0.38
Sirois and Hirsch (2019)	S1: 319 S2:152 S3: 61 S4: 55	UK	Cross‐sectional	S1: Fibromyalgia S2: Fibromyalgia S3: Chronic fatigue S4: Cancer	S1: 47.89 S2: 41.51 S3: 33.91 S4: 61.24	S1: 96.1 S2: 89.4 S3: 83.8 S4: 62.0	–
Sirois, Molnar, and Hirsch (2015)	S1: 155 S2: 170	Canada	Cross‐sectional	S1: Inflammatory bowel disease S2: Arthritis	S1: 38.84 S2: 47.44	S1: 83.1 S1: 91.5	–
Skinta et al. (2019)	90	UK	Cross‐sectional	Human immuno‐deficiency virus	43.5	0	12.24
Snyder et al. (2022)	56	USA	Cross‐sectional	Lung cancer	64.88	100	–
Stutts et al. (2020)	140	USA	Cross‐sectional	Parkinson's disease	68.72	42.1	7.15
Tanenbaum et al. (2018)	542	USA	Cross‐sectional	Type 1 diabetes	41.4	65	23.3
Trindade and Sirois (2021)	155	Portugal	Cross‐sectional/Longitudinal	Inflammatory bowel disease	36.5	69.7	8.54
Unal & Ordu (2023)	151	Turkey	Cross‐sectional	Cancer	54.94	60.9	‐
Van der Donk et al. (2020)	245	Netherlands	Cross‐sectional	Cancer	65.35	24.9	2.39
Van der Heide et al. (2021)	2899	Netherlands	Cross‐sectional	Parkinson's disease	–	–	–
Van Niekerk et al. (2022)	277	Netherlands	Cross‐sectional	Polycystic ovary syndrome	30.57	100	13.19
Vizin et al. (2023)	94	Hungary	Cross‐sectional	Breast cancer	38.8	100	–
Wei et al. (2023)	289	China	Cross‐sectional	Cancer	50.11	0.623	–
Williams et al. (2021)	181	USA	Cross‐sectional	Human immuno‐deficiency virus	42.81	0.249	11.71
Williamson et al. (2022)	108	USA	Cross‐sectional	Lung cancer	64.81	0.481	–
Wren et al. (2012)	88	USA	Cross‐sectional	Chronic pain	53.93	0.716	11.79
Yousefi Afrashteh and Masoumi (2021)	210	Iran	Cross‐sectional	Breast cancer	38.97	100	–
Zhu et al. (2020)	301	China	Cross‐sectional	Cancer	50.07	0.604	1.19
Ziemer (2014)	50	USA	Experimental	Chronic pain	48.9	0.82	–

**TABLE 3:** Meta‐analysed effect sizes for the association of self‐compassion (SC) with psychological distress (PD).

Author (publication year)	*N*	Self‐compassion measure	Psychological distress measure	SC‐PD *r*	95% CI
Abdollahi et al. (2020)	210	SCS	PSS	−.620	[−.697, −.529]
Baker et al. (2019)	270	SCS	HADS	−.597	[−.669, −.514]
Campbell et al. (2022)	108	SCS	DASS, PSS	−.662	[−.756, −.541]
Carvalho et al. (2018)	231	SCS‐SF	DASS	−.550	[−.634, −.453]
Carvalho et al. (2020)	49	SCS	DASS	−.505	[−.688, −.261]
Carvalho et al. (2022)	49	SCS	HADS	−.290	[−.528, −.010]
Costa and Pinto‐Gouveia (2011)	103	SCS	DASS	−.531	[−.657, .376]
Davey et al. (2020)	420	SCS‐SF	PHQ‐9	−.340	[−.422, −.253]
Day (2019)	278	SCS‐SF	PSS	−.690	[−.747, −.623]
Eccles et al. (2023)	130	SCS	DASS	−.630	[−.724, −.514]
Emmerich et al. (2020)	316	SCS‐D	PHQ‐9, PASS	−.460	[−.543, −.368]
Friis et al. (2015)	110	SCS	PHQ‐9, DDS‐2	−.575	[−.688, −.435]
Gedik and Idiman (2020)	89	SCS	HADS	−.430	[−.586, −.244]
Harrison et al. (2017)	70	CORS	IRGL	−.290	[−.661, −.306]
Hirsch et al. (2021)	1218	SCS	PSS, GAD‐2, PHQ‐2	−.421	[−.466, −.374]
Ho et al. (2022)	127	SCS	PANAS, HADS	−.293	[−.445, −.125]
Houston (2022)	147	SCS‐SF	PHQ‐9, GAD‐7	−.456	[−.582, −.309]
Kauser et al. (2021)	114	SCS	DASS	−.485	[−.614, −.331]
Kelliher‐Rabon et al. (2022)	S1:419 S2: 235	SCS‐SF	S1: DASS S2: MHP‐P	S1: −.561 S2: −.582	S1: [−.623, −.492] S2: [−.661, −.491]
Kemppainen et al. (2013)	1969	BVSCI	CES‐D, SCL‐90	−.373	[−.410, −.334]
Kılıç et al. (2022)	116	SCS	PHQ‐8, GAD‐7, PAID	−.543	[−.660, −.400]
Matos‐Pina et al. (2022)	223	SCS	HADS	−.450	[−.549, −.339]
Morgenroth et al. (2022)	45	SCS	PHQ‐9, DDS‐17	−.310	[−.553, −.018]
Morrison et al. (2021)	176	SCS‐SF	K6	−.495	[−.599, −.375]
Ogueji (2021)	832	SCS‐SF	BDI, BAI	−.550	[−.596, −.501]
O'Loughlin et al. (2020)	111	BVSCI	CES‐D, SCL‐90	−.390	[−.537, −.220]
Pinto‐Gouveia et al. (2014)	S1: 68 S2: 63	SCS	DASS	S1: −.521 S2: −.381	S1: [−.676, −.323] S2: [−.575, −.147]
Potter et al. (2020)	144	SCS	DASS	−.510	[−.622, −.378]
Purdie and Morley (2015)	60	SCS	DAPOS	−.618	[−.754, −.432]
Rawlings et al. (2023)	65	SCS	GAD‐7, PHQ‐9	−.395	[−.583, −.167]
Santerre‐Baillargeon et al. (2018)	48	SCS	BDI, STAI	−.565	[−.732, −.335]
Schellekens et al. (2017)	88	SCS‐12	HADS	−.550	[−.681, −.385]
Sirois and Hirsch (2019)	S1: 319 S2:152 S3: 61 S4: 55	SCS	S1: DASS S2 and S3: PSS‐10 S4: PSS‐4	S1: −.583 S2: −.601 S3: −.628 S4: −.625	S1: [−.651, −.506] S2: [−.694, −.489] S3: [−.760, −.447] S4: [−.764, −.431]
Sirois, Molnar, and Hirsch (2015)	S1: 155 S2: 170	SCS	PSS‐10	S1: −.560 S2: −.560	S1: [−.659, −.441] S2: [−.655, −.447]
Skinta et al. (2019)	90	SCS‐SF	CES‐D, STICSA, PANAS	−.618	[−.731, −.471]
Snyder et al. (2022)	56	SCS‐SF	DASS	−.440	[−.630, −.200]
Stutts et al. (2020)	140	SCS	DASS	−.354	[−.491, −.200]
Tanenbaum et al. (2018)	542	SCS‐Diabetes	DDS‐T1	−.610	[−.660, −.554]
Trindade and Sirois (2021)	155	SCS	DASS	−.565	[−.664, −.447]
Unal & Ordu (2023)	151	SCS	BDI, BAI	−.346	[−.479, −.197]
Van der Donk et al. (2020)	245	SCS	CES‐D, PANAS	−.423	[−.521, −.314]
Van der Heide et al. (2021)	2899	SCS‐SF	PSS‐10	−.650	[−.671, −.628]
Van Niekerk et al. (2022)	277	SCS	PROMIS	−.620	[−.694, −.533]
Vizin et al. (2023)	94	SCS	HADS	−.493	[−.632, −.323]
Wei et al. (2023)	289	SCS‐SF	PHQ‐9, STAI‐6	−.244	[−.350, −.132]
Williams et al. (2021)	181	SCS‐SF	CES‐D	−.658	[−.733, −.567]
Williamson et al. (2022)	108	SCS‐SF	CES‐D	−.390	[−.539, −.217]
Wren et al. (2012)	88	SCS	PANAS	−.520	[−.658, −.349]
Yousefi Afrashteh and Masoumi (2021)	210	SCS	BDI, BAI	−.505	[−.599, −.397]
Zhu et al. (2020)	301	SCS‐SF	PHQ‐9, STAI‐6	−.380	[−.473, −.279]
Ziemer (2014)	50	SCS‐SF	CES‐D	−.680	[−.806, −.495]
			Overall effect size	−.516 *K* = 57	[−.549, −.481]

*Note*: ‘–’ indicates data not reported/not obtainable.

Abbreviations: BAI (Beck Anxiety Inventory; Beck et al., 1988), BDI (Beck Depression Inventory; Beck et al., 1961), BVSI (Brief Version of the Self‐compassion Inventory; Kemppainen et al., 2013), CES‐D (Centre for Epidemiologic Studies – Depression Scale; Radloff, 1977), DAPOS (Depression, Anxiety and Positive Outlook Scale; Pincus et al., 2004), DASS (Depression Anxiety and Stress Scale; Lovibond & Lovibond, 1995), DDS‐2 (Diabetes Distress Scale 2‐item, Fisher et al., 2008), DDS‐17 (Diabetes Distress Scale 17‐item; Martinez et al., 2018), DDS‐T1 (Diabetes Distress Scale for Type 1 Diabetes; Fisher et al., 2015), GAD‐2 (Generalized Anxiety Disorder 2‐item; Kroenke et al., 2007), GAD‐7 (Generalized Anxiety Disorder 7‐item; Spitzer et al., 2006), HADS (Hospital Anxiety and Depression Scale; Zigmond & Snaith, 1983), K6 (Kessler Psychological Distress Scale; Kessler et al., 2003), MHP‐P (Multi‐dimensional Health Profile – Part 1; Ruehlman et al., 1999), PAID (Problem Areas In Diabetes Scale, Welch et al., 1997), PANAS (Positive and Negative Affect Scale; Watson et al., 1988), PHQ‐2 (Patient Health Questionnaire 2‐item; Löwe et al., 2005), PHQ‐8 (Patient Health Questionnaire 8‐item; Kroenke et al., 2009), PHQ‐9 (Patient Health Questionnaire 9‐item; Kroenke et al., 2001), PROMIS (Patient Reported Outcomes Measurement Information System; Pilkonis et al., 2011), PSS (Perceived Stress Scale; Cohen et al., 1983), PSS‐2 (Perceived Stress Scale 2‐item), PSS‐10 (Perceived Stress Scale 10‐item; Cohen & Williamson, 1988), S, Sample; SCL‐90 (Symptom Checklist‐90; Derogatis & Cleary, 1977), SCS (Self‐compassion Scale; Neff, 2003a), SCS‐D (Self‐compassion Scale German version, Hupfeld & Ruffieux, 2011), SCS‐Diabetes (Self‐compassion Scale Diabetes Specific Version; Tanenbaum et al., 2018), SCS‐SF (Self‐compassion Scale Short Form; Raes et al., 2011), STAI (State–Trait Anxiety Index; Spielberger, 1970), STAI‐6 (State Trait Anxiety Inventory 6‐item; Marteau & Bekker, 1992), STICSA (State Trait Inventory for Cognitive and Somatic Anxiety; Grös et al., 2007).

### Quality assessment

All studies included in the meta‐analysis were of either moderate or high quality (see Appendix B: Data S1). A second researcher independently assessed a subset of the papers (*k* = 15). Inter‐rater agreement was initially at 80% and increased to 100% following discussions. The highest quality score was 10 (*k* = 19) the lowest was 8 (*k* = 9), and the remaining studies were rated as moderate or high in quality.

### Overall meta‐analysis

Of the 57 effects included in the analysis (see Table 3), 53 were *r* values and 4 were *p* values (Spearman's rank correlation co‐efficient). Given the similarity between these metrics, conversion to a common metric was not required. As expected, there was a significant large negative association between self‐compassion and psychological distress outcomes (*r* = −.516; 95% CIs [−.55, −.48]; *z* = −24.257, *p* = .000). There was evidence of high heterogeneity *Q*(56) = 390.475, *p* = .000, *I* ^2^ = 85.66%, indicating that moderator analyses were warranted.

### Sensitivity analyses

Sensitivity analysis revealed that removing the four studies that reported Spearman's rank rather than Pearson's correlation co‐efficient (Baker et al., 2019; Eccles et al., 2023; Morrison et al., 2021; Van Niekerk et al., 2022), did not largely impact the overall effect size (*r* = −.509, *k* = 53; 95% CIs [−.54, −.47]; *z* = −22.63, *p* = .000), supporting inclusion of these studies in the meta‐analysis. Similarly, removal of the one study (Harrison et al., 2017) that did not use a variant of the self‐compassion scale yielded effects that were almost identical to the original (*r* = −.516; 95% CIs [−.55, −.48]; *z* = −24.077, *p* = .000).

### Moderator analyses

Effect sizes were grouped according to the type of psychological distress measured, resulting in three subgroups; stress (*k* = 8; *N* = 4021), depression (*k* = 11; *N* = 1834) and overall distress (*k* = 38; *N* = 9569). There was an insufficient number of effects to create a distinct subgroup for anxiety. The subgroup analysis indicated that the effects obtained from studies that measured stress (*r* = −.618, 95% CI [−.65, −.59], *p* = .000), depression (*r* = −.527, 95% CI [−.61, −.44], *p* = .000), and overall distress (*r* = −.491, 95% CI [−.43, −.45], *p* = .000) differed significantly in magnitude (*Q*(2) = 27.94, *p* = .000). Studies that measured distress as stress had larger effects than those that measured it as symptoms of depression or overall distress.

Grouping illness types resulted in six moderator subgroups; cancer (*k* = 14; *N* = 2232), endocrine (*k* = 5; *N* = 1171), HIV (*k* = 5; *N* = 3183), neurological (*k* = 7; *N* = 3914), pain (*k* = 14; *N* = 3643) and ‘other’ (*k* = 12; *N* = 1281), which included illnesses that could not be categorized into distinct subgroups. The analysis revealed that the effects obtained from studies that measured cancer (*r* = −.452, 95% CI [−.52, −.38], *p* = .000), endocrine (*r* = −.583, 95% CI [−.63, −.54], *p* = .000), HIV (*r* = −.523, 95% CI [−.63, −.40], *p* = .000), neurological (*r* = −.593, 95% CI [−.66, −.52], *p* = .000), pain (*r* = −.525, 95% CI [−.58, −.47], *p* = .000), and other (*r* = −.493, 95% CI [−.54, −.45], *p* = .000) illness types, differed significantly in magnitude (*Q*(5) = 16.413, *p* = .006). Studies that measured distress in participants with neurological conditions had the largest average effects and those that measured distress in participants with cancer had the smallest average effects.

Meta‐regressions revealed that the magnitude of the associations between self‐compassion and psychological distress did not differ as a function of participant sex (*Q*(1) = 1.354, *b* = −.115, *p* = .0598, 95% CI [−.36, .13], *z* = −.90), age (*Q*(1) = 1.05, *b* = −.003, *p* = .366, 95% CIs [−.003, .001], *z* = 1.02), or illness duration (*Q*(1) = 1.39, *b* = −.008, *p* = .238, 95% CIs [−.022, .005], *z* = −1.18).

### Tests of publication bias

The tests suggested minimal publication bias. Fail‐safe *N* (Rosenthal, 1979) revealed that 53,545 studies with null results would need to be included in the analysis for the effects to become no longer be significant, surpassing the Fail‐safe *N* threshold of 295 (5 *k +* 10). Visual inspection of the funnel plots indicated of minor asymmetry around the mean effect size, confirmed by the trim‐and‐fill method which imputed one study to the right of the mean. With the additional study imputed, the pooled effect size was slightly larger but overall similar to the original effect size (*r* = .529, 95% CIs [−.56, −.50]). Egger's regression test was also non‐significant (*t*(55) = .683, *p* = .497).

## DISCUSSION

The current meta‐analysis is the first comprehensive quantitative investigation of the association between self‐compassion and psychological distress in different chronic illness populations, and evaluation of the factors that moderate this association. Our analysis of 57 effects from a pooled sample of 15,424 people with various chronic illnesses found that self‐compassion was strongly associated with lower psychological distress. As expected, we found that the strength of this association varied according to the way that distress was measured, with studies assessing distress as stress reporting a stronger link between self‐compassion and distress than those where distress was measured as symptoms of depression or as multiple types of distress. Similarly, the protective role of self‐compassion for distress varied according to the type of chronic illness. However, age, gender, and illness duration did not influence the magnitude of the link between self‐compassion and psychological distress.

The large association between self‐compassion and psychological distress found in the current meta‐analysis (*r* = −.52) is comparable to the large effect size (*r* = −.54) found in a meta‐analysis examining the association between self‐compassion and psychopathology in general adult samples (Macbeth & Gumley, 2012). In that meta‐analysis, only 20 effects were analysed with a pooled sample size of 4007 adults. That we found similar results when examining distress in a much larger pool of studies and in people living with a chronic illness, highlights the importance of self‐compassion as a protective positive psychological quality for reducing psychological distress, whether in chronically ill or healthy adult populations.

One noteworthy finding from the moderator analyses is that self‐compassion may be especially protective for people with a chronic illness when psychological distress is experienced as stress. Individuals living with chronic health conditions are susceptible to experiencing significant illness‐related stressors (Sirois, Kitner, & Hirsch, 2015), which can contribute to disease relapse, progression, and pain (Evers et al., 2013; Jaghulta et al., 2013; Maunder, 2005). This reciprocal relationship means that stress can be particularly detrimental to psychological and physical well‐being of those with a chronic illness. Previous research indicates that self‐compassion reduces stress in chronic illness populations by supporting engagement in adaptive coping strategies and health‐promoting behaviours (Sirois, 2015; Sirois & Hirsch, 2019; Sirois, Molnar, & Hirsch, 2015). Our findings are consistent with this evidence and indicate that self‐compassion is one positive psychological quality that reduces the burden of stress in chronic illness populations.

Similar to previous research comparing the association of self‐compassion with psychological distress between chronic illness groups (Pinto‐Gouveia et al., 2014), we found that the strength of this association differed across chronic illness types. Specifically, the magnitude was the strongest for neurological conditions, and weakest for cancer. This may be due to differences in the types of challenges that characterize these conditions, such as functional limitations and reduced independence (Mistretta & Davis, 2022; Sirois, Kitner, & Hirsch, 2015). Crucially, longitudinal research indicates that stress in turn has a bidirectional, negative relationship with functionality in multiple sclerosis (Aragonès et al., 2023), suggesting a vicious cycle between stress and functionality. Ostensibly, it could also be that the impact of self‐compassion on distress was attenuated in cancer patients due to the life‐threatening nature of cancer. However, as the 13 cancer patient samples included a variety of cancer types (see Table 1), and disease stages (i.e. 6 with various stages, 1 stage 4, and 6 that did not report cancer stage), drawing this conclusion would be highly speculative. Further research investigating the possible reasons for these moderation effects is necessary to better understand the differences in effects among different chronic conditions.

The magnitude of the association between self‐compassion and psychological distress was also relatively consistent across different ages, genders and illness duration. This finding is consistent with the previous meta‐analysis conducted in the general adult population, which also did not find that the association between self‐compassion and psychological distress varied according to participant sex and age (Macbeth & Gumley, 2012). This suggests that self‐compassion is a useful resource for reducing psychological distress in chronic illness populations regardless of these individual differences.

Although the type of psychological distress measured and the chronic illness type explained a degree of the heterogeneity in the pooled effects, some remained unexplained. It is not always possible to identify all the factors that contribute to differences between samples (Riley et al., 2011). Nonetheless, one possible source of heterogeneity that was not examined is disease severity. More severe symptomology has been linked to a pessimistic illness perspective, which in turn increased psychological distress in certain illness groups (Zhang et al., 2016). Given the links between self‐compassion and more positive appraisals of chronic illness difficulties (e.g. Morgenroth et al., 2022; Pinto‐Gouveia et al., 2015), differences in levels of disease severity across studies could account for some of the unexplained variance in the associations between self‐compassion and psychological distress. Recent research examining self‐compassion in people with chronic pain also found that stigma was an important contributor to chronic pain outcomes, including distress (Anderson et al., 2024). It is therefore possible that differences in stigma as a result of chronic illness may also explain some of the variance between self‐compassion and psychological distress.

### Strengths and limitations

The current findings should be considered in light of several limitations and strengths. The cross‐sectional design of the studies analysed means that causation cannot be established. However, self‐compassion theory and research proposes that self‐compassion reduces psychological distress through addressing ruminative thoughts, promoting adaptive reappraisal of challenges and struggles, and supporting adaptive coping (Johnson & O'Brien, 2013; Sirois, Molnar, & Hirsch, 2015). Supporting this proposition, research has found that self‐compassion interventions are effective for reducing psychological distress in people with diabetes (Friis et al., 2016), and youth with chronic medical conditions (Finlay‐Jones et al., 2023). Further longitudinal research would nonetheless help to clarify the proposed temporal sequencing of the links between self‐compassion and psychological distress.

Although we examined several potential moderators, including the type of distress, the small numbers of studies examining anxiety symptoms alone meant that moderator analysis of this form of distress was not possible. Instead, anxiety was subsumed within the “other” distress category which reflected the composite associations of self‐compassion with multiple forms of distress. Further research is needed to clarify the extent to which anxiety is associated with self‐compassion. Due to the diverse range of chronic health conditions across the pool of studies analysed, different chronic illnesses were categorized into subgroups based on both similarities in symptomology (e.g. pain) and cause (for example, hormone dysfunction). This resulted in relatively small subgroups being compared (i.e. *k* = 5 for endocrine and HIV), as well as a subgroup that included a diverse range of illnesses that did not necessarily have commonalities. It is therefore possible that a different categorisation scheme would have resulted in different findings for the moderator analysis of illness type.

We included a large number of descriptors of chronic illness in our search strategy; however, it is possible that some were not included. For example, we included fibromyalgia and chronic pain, but did not include more specific subtypes of chronic pain such as back pain, or neuropathic pain, or noci‐plastic pain for which self‐compassion may be relevant with regards to distress. Whilst it is possible that some of these specific types of chronic pain may have been captured under the broader search term of “chronic pain”, it is also possible that they were not and therefore their effects are not represented in the analyses.

A key strength of this meta‐analysis is the large number of effects identified for inclusion (57) and large overall sample size from the studies included (*N* = 15,424), with more than two thirds of the included studies having a sample size of over 100. This suggests that the majority were sufficiently powered to detect a medium‐sized correlation (Cohen, 1992). Multiple tests of publication bias were used and indicated any bias was minimal. High inter‐rater reliability was established for both the data extraction and quality appraisal processes. A further strength is the use of sensitivity analyses to examine the impact of methodological differences on the pooled effect size. The sensitivity analyses did not result in a large change in the effect size, indicating that it was robust to these methodological differences, thus increasing the reliability and validity of the findings. Lastly, the quality analysis found that all studies were rated as high or moderate in quality, increasing confidence that the effect size estimates are reliable and valid.

### Future directions and clinical implications

This meta‐analysis found a robust relationship between self‐compassion and lower psychological distress, highlighting the importance of further understanding the mechanisms that link these two variables. Research has begun to identify some of these mechanisms; for example, the use of more adaptive coping styles in those with inflammatory bowel disease and arthritis for reducing stress (Sirois, Molnar, & Hirsch, 2015), and the ability to take a decentred perspective of illness in those with heart failure (Morgenroth et al., 2022). Further investigation of these and other mechanisms that link self‐compassion and psychological distress, across various chronic illness groups, is warranted. Such research would improve understandings of why individuals with a chronic illness who are more self‐compassionate experience less psychological distress, help identify chronic illness groups most vulnerable to having lower self‐compassion, and inform interventions that aim to improve self‐compassion in people with chronic illness.

The evidence‐base for interventions that target self‐compassion in chronic illness populations is rapidly evolving. Interventions based on Compassion Focused Therapy (CFT) are considered to be effective in supporting those with chronic health conditions to reduce self‐criticism that encourages striving behaviours and ultimately, worsening of illness symptomology (Malpus et al., 2023). Acceptance and Commitment Therapy (ACT) is another therapy with a self‐compassion component and has been recommended by the National Institute for Health and Care Excellence (NICE) for treatment of chronic pain (NICE, 2021). ACT is thought to improve self‐compassion by increasing acceptance and non‐judgemental awareness of negative thoughts and feelings (Neff & Tirch, 2013).

The magnitude of the association between self‐compassion and psychological distress found in this meta‐analysis, and the links between self‐compassion and acceptance of difficulty in chronic illness populations, suggests that further investigation of self‐compassion‐based interventions in treatment of chronic health conditions is warranted. A recent meta‐analysis found small effects of self‐compassion focused interventions on self‐compassion in individuals with chronic health conditions and psychological difficulties; however, the included studies were of poor quality (Mistretta & Davis, 2022). A systematic review of the effectiveness of self‐compassion‐related interventions in people with chronic physical conditions found that such interventions were effective for increasing self‐compassion and improving psychological outcomes (Kılıç et al., 2021). Similarly, a mixed methods systematic review found that compassion‐based interventions were effective for reducing depression and anxiety (Austin et al., 2021). Further research examining the efficacy of self‐compassion‐based interventions in supporting individuals with chronic health conditions that uses rigorous methodologies, such as randomized control trials, is therefore needed.

## CONCLUSIONS

In the current meta‐analysis self‐compassion was strongly associated with lower psychological distress in individuals with chronic illness. This large‐sized association was robust to the influence of age, participant sex, and illness duration. However, the magnitude of this association varied significantly depending on the way in which distress was assessed, and on the type of chronic illness population. Research focusing on understanding the underlying mechanisms that link self‐compassion and psychological distress, and that examine the effectiveness of self‐compassion interventions in chronic illness populations, is needed to further advance knowledge and inform practice in this area. Such research would provide insights into the implications of being more self‐compassionate in the face of chronic illness difficulties and improve understandings of how to increase self‐compassion to help reduce psychological distress in those living with a chronic illness.

## AUTHOR CONTRIBUTIONS

**Rebecca Baxter:** Conceptualization; investigation; writing – original draft; formal analysis; data curation; writing – review and editing; project administration. **Fuschia M. Sirois:** Conceptualization; formal analysis; supervision; writing – review and editing; data curation.

## Supporting information


Data S1.

## Data Availability

As this is a meta‐analysis of published studies there is no original data to share.
