# Supplementary material for: Self‐compassion and psychological distress in chronic illness: A meta‐analysis
Source: Br J Health Psychol. 2024 Nov 7;30(1):e12761. doi: 10.1111/bjhp.12761 (PMC11586813; doi:10.1111/bjhp.12761)
Supplement: Supplementary file 1 — Data S1. [file BJHP-30-0-s001.docx]

Appendix A: Quality Appraisal Criteria

1. Were the hypotheses/aims/objectives of the study clear?
2. Was the method of obtaining the data clearly described?
3. Were criteria for inclusion in the sample clearly defined?
4. Was the target/reference population clearly defined?
5. Was the sample taken from an appropriate population base so that it closely represented the target/reference population under investigation?
6. Was the selection process likely to select participants that were representative of the target/reference population under investigation (i.e., random sampling)?
7. Were the outcome variables measured using validated and reliable means?
8. Was the independent variable measured using validated and reliable means?
9. Was appropriate statistical analysis used?
10. Were the methods (including statistical methods) sufficiently described to enable them to be repeated?
11. Did the study describe any limitations?

Appendix B: Quality assessment

|  | **Quality criteria** | | | | | | | | | | | |
| --- | --- | --- | --- | --- | --- | --- | --- | --- | --- | --- | --- | --- |
| **Author (Year)** | **1** | **2** | **3** | **4** | **5** | **6** | **7** | **8** | **9** | **10** | **11** | **Total** |
| Abdollahi et al. (2020) | 1 | 1 | 0 | 1 | 1 | 0 | 1 | 1 | 1 | 1 | 1 | 9 |
| Afrashteh & Masoumi (2021) | 1 | 1 | 1 | 1 | 1 | 0 | 1 | 1 | 1 | 1 | 1 | 10 |
| Baker et al. (2019) | 1 | 1 | 1 | 1 | 0 | 0 | 1 | 1 | 1 | 1 | 1 | 9 |
| Campbell et al. (2022) | 1 | 1 | 1 | 1 | 0 | 0 | 1 | 1 | 1 | 1 | 1 | 9 |
| Carvalho et al. (2018) | 1 | 1 | 1 | 1 | 0 | 0 | 1 | 1 | 1 | 0 | 1 | 8 |
| Carvalho et al. (2020) | 1 | 1 | 1 | 1 | 0 | 0 | 1 | 1 | 1 | 0 | 1 | 8 |
| Carvalho et al. (2021) | 1 | 1 | 1 | 1 | 0 | 0 | 1 | 1 | 1 | 1 | 1 | 9 |
| Costa & Pinto-Gouveia (2011) | 1 | 1 | 1 | 1 | 1 | 0 | 1 | 1 | 1 | 1 | 1 | 10 |
| Davey et al. (2020) | 1 | 1 | 0 | 1 | 1 | 0 | 1 | 1 | 1 | 1 | 1 | 9 |
| Day (2019) | 1 | 1 | 1 | 1 | 1 | 0 | 1 | 1 | 1 | 1 | 1 | 10 |
| Eccles et al. (2023) | 1 | 1 | 1 | 1 | 0 | 0 | 1 | 1 | 1 | 1 | 1 | 9 |
| Emmerich et al. (2020) | 1 | 1 | 1 | 1 | 1 | 0 | 1 | 1 | 0 | 0 | 1 | 8 |
| Friss et al. (2015) | 1 | 1 | 1 | 1 | 1 | 0 | 1 | 1 | 1 | 1 | 1 | 10 |
| Gedik & Idiman (2020) | 1 | 1 | 1 | 1 | 1 | 0 | 1 | 1 | 1 | 1 | 1 | 10 |
| Harrison et al. (2017) | 1 | 1 | 1 | 1 | 1 | 0 | 1 | 1 | 1 | 1 | 1 | 10 |
| Hirsch et al. (2021) | 1 | 1 | 0 | 1 | 1 | 0 | 1 | 1 | 1 | 0 | 1 | 8 |
| Ho et al. (2022) | 1 | 1 | 1 | 1 | 1 | 0 | 1 | 1 | 1 | 1 | 1 | 10 |
| Houston (2022) | 1 | 1 | 1 | 1 | 1 | 0 | 1 | 1 | 1 | 1 | 1 | 10 |
| Kauser et al. (2021) | 1 | 1 | 0 | 1 | 1 | 0 | 1 | 1 | 1 | 1 | 1 | 9 |
| Kelliher-Rabon et al. (2021) | 1 | 1 | 0 | 1 | 0 | 0 | 1 | 1 | 1 | 1 | 1 | 8 |
| Kemppainen et al. (2013) | 1 | 1 | 1 | 1 | ? | 0 | 1 | 1 | 1 | 0 | 1 | 8 |
| Kılıç et al. (2022) | 1 | 1 | 1 | 1 | 0 | 0 | 1 | 1 | 1 | 1 | 1 | 9 |
| Matos-Pina et al. (2023) | 1 | 1 | 0 | 1 | ? | 0 | 1 | 1 | 1 | 1 | 1 | 8 |
| Morgenroth et al. (2022) | 1 | 1 | 1 | 1 | 1 | 0 | 1 | 1 | 1 | 1 | 1 | 10 |
| Morrison et al. (2019) | 1 | 1 | 1 | 1 | 1 | 0 | 1 | 1 | 1 | 1 | 0 | 9 |
| Ogueji (2021) | 1 | 1 | 1 | 1 | 1 | 0 | 1 | 1 | 1 | 1 | 0 | 9 |
| O'Loughlin et al. (2020) | 1 | 1 | 0 | 1 | ? | 0 | 1 | 1 | 1 | 1 | 1 | 8 |
| Pinto-Gouveia et al. (2014) | 1 | 1 | 1 | 1 | 1 | 0 | 1 | 1 | 1 | 0 | 1 | 9 |
| Potter et al. (2020) | 1 | 1 | 1 | 1 | 0 | 0 | 1 | 1 | 1 | 1 | 1 | 9 |
| Purdie & Morley (2015) | 1 | 1 | 1 | 1 | 1 | 0 | 1 | 1 | 1 | 1 | 1 | 10 |
| Rawlings et al. (2023) | 1 | 1 | 1 | 1 | 1 | 0 | 1 | 1 | 1 | 1 | 1 | 10 |
| Santerre-Baillargeon et al. (2018) | 1 | 1 | 1 | 1 | 0 | 0 | 1 | 1 | 1 | 1 | 1 | 9 |
| Schellekens et al. (2017) | 1 | 1 | 1 | 1 | ? | ? | 1 | 1 | 1 | 1 | 1 | 9 |
| Sirois & Hirsch (2019) | 1 | 1 | 0 | 1 | 0 | 0 | 1 | 1 | 1 | 1 | 1 | 8 |
| Sirois et al. (2015) | 1 | 1 | 1 | 1 | 0 | 0 | 1 | 1 | 1 | 1 | 1 | 9 |
| Skinta et al. (2018) | 1 | 1 | 1 | 1 | 1 | 0 | 1 | 1 | 1 | 1 | 1 | 10 |
| Snyder et al. (2022) | 1 | 1 | 1 | 1 | 0 | 0 | 1 | 1 | 1 | 1 | 1 | 9 |
| Stutts et al. (2020) | 1 | 1 | 1 | 1 | ? | ? | 1 | 1 | 1 | 1 | 1 | 9 |
| Tanenbaum et al. (2018) | 1 | 1 | 1 | 1 | 0 | 0 | 1 | 1 | 1 | 1 | 1 | 9 |
| Trinidade & Sirois (2021) | 1 | 1 | 0 | 1 | 1 | 0 | 1 | 1 | 1 | 1 | 1 | 9 |
| Unal & Ordu (2023) | 1 | 1 | 1 | 1 | 1 | 0 | 1 | 1 | 1 | 1 | 1 | 10 |
| Van der Donk et al. (2020) | 1 | 1 | 0 | 1 | 1 | 0 | 1 | 1 | 1 | 1 | 1 | 9 |
| Van der Heide et al. (2021) | 1 | 1 | 1 | 1 | 1 | 0 | 1 | 1 | 1 | 1 | 1 | 10 |
| Van Niekirk et al. (2022) | 1 | 1 | 1 | 1 | 1 | 0 | 1 | 1 | 1 | 0 | 1 | 9 |
| Vizin et al. (2023) | 1 | 1 | 1 | 1 | 1 | 0 | 1 | 1 | 1 | 1 | 1 | 10 |
| Wei et al. (2022) | 1 | 1 | 1 | 1 | 0 | 0 | 1 | 1 | 1 | 1 | 1 | 9 |
| Williams et al. (2021) | 1 | 1 | 1 | 1 | 1 | 0 | 1 | 1 | 1 | 1 | 1 | 10 |
| Williamson et al. (2022) | 1 | 1 | 1 | 1 | 0 | 0 | 1 | 1 | 1 | 1 | 1 | 9 |
| Wren et al. (2012) | 1 | 1 | 1 | 1 | 1 | 0 | 1 | 1 | 1 | 1 | 1 | 10 |
| Zhu et al. (2020) | 1 | 1 | 1 | 1 | 1 | 0 | 1 | 1 | 1 | 1 | 1 | 10 |
| Ziemer (2014) | 1 | 1 | 1 | 1 | 1 | 0 | 1 | 1 | 1 | 1 | 1 | 10 |

*Notes.* 1 = Meets criteria, 0 = does not meet criteria, ? = not clear from the paper.
